# Tubule and microbranch variations in human dentine: A quantitative 3D study with serial block‐face scanning electron microscopy

**DOI:** 10.1111/jmi.70123

**Published:** 2026-05-22

**Authors:** Bethany Harding, Patricia Goggin, James Thompson, Behrad Mahmoodi, Katy Rankin, Philipp Schneider, Richard Cook

**Affiliations:** ^1^ National Centre for Advanced Tribology at Southampton (nCATS) Faculty of Engineering and Physical Sciences University of Southampton Southampton UK; ^2^ Bioengineering Science Research Group Faculty of Engineering and Physical Sciences University of Southampton Southampton UK; ^3^ Biomedical Imaging Unit University Hospital Southampton NHS Foundation Trust Southampton UK; ^4^ Oral Health R&D Haleon plc Weybridge Surrey UK; ^5^ μ‐VIS X‐Ray Imaging Centre Faculty of Engineering and Physical Sciences University of Southampton Southampton UK; ^6^ UK Science & Technology Network British Embassy Berne Bern Switzerland

**Keywords:** dentine microbranches, dentine tubules, serial block face scanning electron microscopy, volume electron microscopy

## Abstract

**Objective**: Microbranches exist within dentine, providing a network of fluid filled connections between tubules. This study used serial block‐face scanning electron microscopy to generate 3D volumes of dentine from different tooth locations, allowing for investigations of the microbranch morphology, the microbranch network characteristics and regional variations in the tooth.

**Methods**: Three human teeth were sectioned to generate 21 dentine samples extracted from 7 identical regions of interest around the tooth. Serial block‐face scanning electron microscopy was performed using a pixel size of 10 nm, a slice thickness of 50 nm, and a field of view of 20.48 µm × 20.48 µm. Tubules and microbranches were automatically segmented and the following quantified: tubule diameter and density; microbranch diameter, density, and connectivity; tissue porosity.

**Results**: The microbranches were successfully captured at sufficient resolution to create a 3D volume from sequential 2D images. The microbranches move tortuously through the intertubular dentine and travelled a variety of distances before connecting to other tubules. The microbranch density varied by location, with more found in areas of lower tubule density. Microbranches significantly increased tissue porosity (*p* < 0.001). Microbranch diameter did not vary regionally, with most approximately 140–210 nm, regardless of sample location. Tubule density and diameter varied radially—both largest closest to the pulp cavity, then decreasing to the dentinoenamel junction.

**Conclusions**: This study provides new three‐dimensional insight into the morphology and network of the dentinal microbranch network, demonstrating a variability in the density of the microbranches with location around the tooth and a significant increase in tissue porosity.

## 1 INTRODUCTION

Fluid movement through dentinal tubules plays an important role in the two main structural functions of the tissue: pressure distribution and mechanotransduction. It also serves to provide a protective barrier against the ingress of oral bacteria, as it is subject to consistent outward pressure.^1^ The fluid movement within dentinal tubules is governed by the Hydrodynamic Theory, with non‐physiologically normal levels of fluid flow resulting in clinical problems such as dentine hypersensitivity.^2^, ^3^, ^4^ The tubule anatomy and structure, including the number of tubules (tubule density) and their diameter, has previously been captured predominantly using 2D scanning electron microscopy (SEM).^1^, ^5^, ^6^, ^7^ The anatomy and function of single tubules is well accepted; the tubule density is reduced away from the pulp cavity,^1^, ^5^, ^8^ as is diameter, varying from approximately 2.5–3 µm at the pulp cavity to 0.8–0.9 µm at the dentine‐enamel junction (DEJ).^5^, ^6^, ^7^, ^8^ However, the tubules are not isolated tubes but connected via a network of microbranches that divert from the main tubules and permeate through the intertubular dentine (ITD) tissue. Several theories exist as to the purpose of this microbranch network, which all only refer to it during dentinogenesis. Mainly, these are: the promotion of intercellular communication,^9^ traces of the branched odontoblast cellular extensions,^6^, ^7^, ^10^ or the need to sense stresses and strains at the DEJ during the first stages of dentinogenesis and mineralisation.^9^ However, little is known of the implications of this network after this stage, and the influence they may have in the regular function of the dentine tissue.

Microbranches were first quantified and categorised by diameter by Mjör and Nordahl.^10^ They found lateral branches with diameters between 0.025–1.0 µm using 2D light microscopy and SEM, dividing these further into ‘Major branches’ (0.5–1.0 µm diameter), ‘Fine branches’ (0.3–0.7 µm diameter), and ‘Micro branches’ (0.025–0.2 µm diameter). However, only a few attempts have been made since to characterise these in 3D, and this has not been on a large scale. Further work was performed by Vennat et al.,^9^, ^11^ who using confocal laser scanning microscopy (CLSM), corroborated the findings above, measuring pores of approximately 0.5–0.75 µm in diameter in dentine close to the DEJ. Further quantification of the porosity of the tissue was also done, and the presence of the microbranches accounted for up to 30% of the tissue's porosity.^9^, ^12^

The movement of fluid through the dentine tubules is key to the healthy function of the tissue. However, when considering the porosity variations seen, connectivity between nearby or neighbouring tubules would be an important consideration influencing both fluid movement and stimuli transfer. Despite being commented upon,^9^, ^12^, ^13^ little is known quantitatively about the potential degree of interconnectivity between adjacent or nearby tubules through these microbranches. This may also have a clinical effect with issues such as dentine hypersensitivity arising from excessive fluid movement in the tissue. With treatment relying on the occlusion of exposed tubules at their surface,^14^, ^15^, ^16^, ^17^ sub‐surface connectivity between nearby or neighbouring tubules still allowing the movement of fluid may limit the efficacy of such treatments. Greater understanding of this combined network or tubules and microbranches together may help with improving clinical treatments.

Serial block‐face (SBF) SEM is a 3D volumetric imaging technique that builds a *z*‐stack of images by cyclically imaging a block face and removing a nanometre‐scale slice from the surface, as showcased by Goggin et al.^18^ for bone tissue. The benefits of SBF SEM over other 3D techniques include higher spatial resolution than computed tomography, and larger fields of view and z‐depth potential than focussed ion beam (FIB) SEM.^8^, ^13^, ^19^, ^20^

The purpose of this study was to use SBF SEM to perform high‐resolution 3D imaging on samples of human dentine and assess their structure and anatomical properties. The size of the microbranches and potential regional variations around the tooth have not been thoroughly investigated in 3D; the goal of this work was to investigate microbranch morphology and for the first time, assess their ability to connect the main tubules as part of a larger network.

## 2 MATERIALS AND METHODS

### 2.1 Sample preparation

**FIGURE 1:**
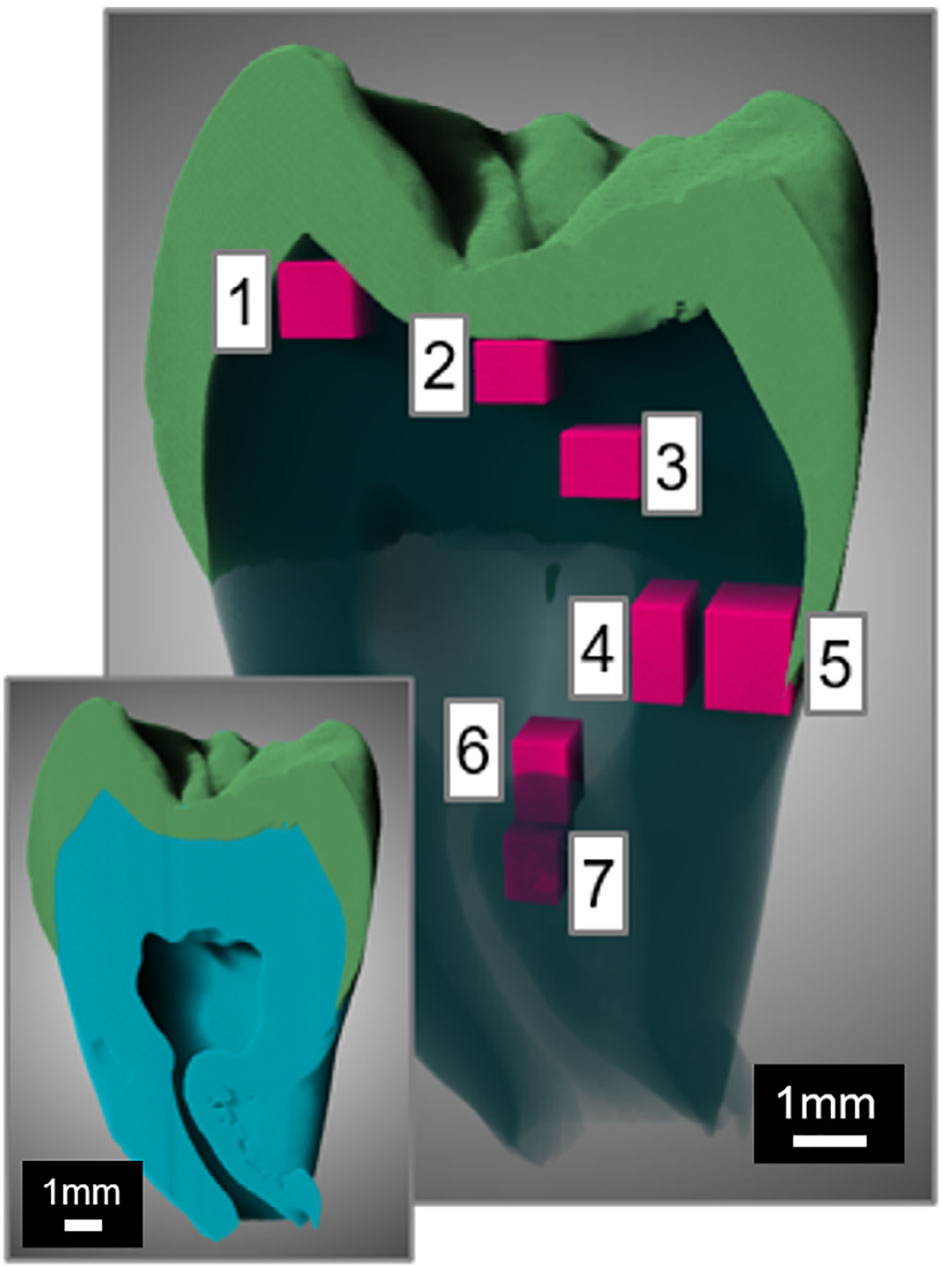
Example sample locations used in this study (inset: Computed tomography scan of Tooth #1 used for representation). Locations are denoted as follows: (1) under cusp, (2) under fissure, (3) bulk coronal, (4) transitional pulp, (5) transitional periphery, (6) upper root, (7) lower root.

Three healthy, human molars were supplied by Modus Laboratories (Plymouth, UK), under ethical approval from the University of Southampton (ERGO approval number: 55910). The molars were supplied by patients in their 20s and were pre‐sectioned in half along the mesiodistal plane to allow for the full removal of all pulpal tissue, as per the ethics agreement. Three teeth were evaluated in this study to remove the effects of interdental variety on the results. Seven regions of interest were chosen for in‐depth visualisation, based on previous 2D observations of tubule structure variation.^1^, ^5^, ^6^, ^8^, ^10^ Regional variations were observed, including higher tubule density and diameter near the pulp cavity compared to near the dentinoenamel or dentinocementum junctions, and higher tubule density in the crown of the tooth compared to the root. The chosen regions are listed in Table 1 using their assigned sample descriptors; a diagram showing their location relative to the whole tooth anatomy is shown in Figure 1. These same locations were identified and sectioned in all three teeth.

**TABLE 1:** Chosen regions of interest for study, with designated names and descriptions of location. Location number corresponds to Figure 1.

Location number	Location name	Location description
1	Under cusp	Directly below one of the cusps, as close as could be reasonably sectioned to the dentinoenamel junction
2	Under fissure	Directly below the central ridge of the biting surface
3	Bulk coronal	In the approximate centre of the tooth crown, far from the dentinoenamel junction or pulp cavity
4	Transitional pulp	In the transitional region between crown and root dentine, directly next to the pulp cavity
5	Transitional periphery	In the transitional region between crown and root dentine, directly next to the dentinoenamel/dentinocementum junction
6	Upper root	In one of the tooth roots, slightly below the transitional zone
7	Lower root	In the same tooth root as upper root, as close to the root apex as could be reasonably sectioned

Sectioning was performed using an Isomet™ low speed saw [Buehler, Illinois, USA) to approximately 1 mm^3^. These were air‐dried, embedded in low viscosity Spurr‐type resin^21^ (ERL 4221, Agar Scientific Ltd., Stansted, UK) and polymerised at 60°C for 16 h. The use of elevated temperature to embed calcified tissue in resin is a common technique, demonstrated by Goggin et al.^18^ to establish the use of SBF SEM on bone tissue—there has been no indication of heat‐induced tissue damage in calcified tissue at this temperature. Once set, the dentine block and excess resin were trimmed using a Leica UC7 microtome (Leica Microsystems, Wetzlar, Germany) and transferred to a G312/1 aluminium pin (TAAB Laboratories Equipment ltd., Aldermaston, UK) using conductive glue (CW2400 conductive epoxy adhesive, Chemtronics, Georgia, USA), being careful to preserve sample orientation. The tissue had not been decalcified and no heavy metal staining was carried out, so the pins were sputter coated with a layer of gold and palladium (Polaron E5100, Quorum Technologies, Laughton, UK) to increase conductivity.

### 2.2 SBF SEM

The samples were imaged using a 3View^®^ 2XP (Gatan UK, Abingdon, UK) attachment on an FEI Quanta 250 field emission gun scanning electron microscope (Thermo Fisher Scientific, Eindhoven, Netherlands). A vacuum level of 20 Pa was used, and an accelerating voltage of 3 kV. The chosen image size was 2048 × 2048 pixels, with a 10 nm pixel size and a dwell time of 5 µs, resulting in a field of view of 20.48 µm × 20.48 µm. Each slice was 50 nm thick, and a minimum of 350 slices were taken from each sample, resulting in a minimum stack height of 17.5 µm.

It should be noted that no further quantification could be made on the Lower Root sample of the second tooth, as the imaged field of view was covered almost entirely by interglobular dentine, and so was deemed unrepresentative, and removed from further assessment.

### 2.3 Image processing

The following steps were performed using (Fiji is Just) ImageJ 2.1.0/1.53c. Pre‐processing steps were necessary to tidy the image stacks. Erroneous images, containing visible debris, were removed. This is an artefact of the serial sectioning procedure and occurred on single slices only. The relative depth of each slice (50 nm) to the entire stack depth (> 17.5 µm) meant these could be removed without significantly altering the final model geometry or appearance. Stack misalignment was also rectified at this stage. The stack was split, the distance moved measured by identifying individual or groups of tubules before and after the movement, and the latter portion of the stack shifted using a rigid translation of a fixed number of pixels using ImageJ, so it is in line with the former. This movement was always less than the image width (< 20.48 µm), as it was possible to identify specific tubules to aid with realignment. Due to its combined fibrous and crystalline texture, the ITD also contained noise (high brightness variations localised to single or small numbers of pixels), so a 3‐pixel median filter was applied to all images, to smoothen and remove this noise prior to segmentation. Both Median and Gaussian filters ranging in size from 1 to 5 pixels were evaluated, and the median filter offered the best visual reduction in noise without losing the detail of the microbranches or tubules.

Automatic segmentation is a process whereby pixels are labelled based on their value by a trained classifier algorithm, assigning them a new binary value. Training was performed using a two‐class trainable Weka classifier, available in Fiji ImageJ.^22^ This was trained on 10‐slice sections from the centre of the first two regions of interest imaged, by manually assigning the pixels into two classes: ITD; and tubules and microbranches. Once trained, this classifier was applied to all other regions of interest slice‐by‐slice using a macro adapted from the ImageJ wiki.^23^ Briefly, this macro allowed for the application of a previously trained classifier to large datasets, slice‐by‐slice without manual input. This classifier was validated against a ground truth case, wherein BH (the first author of this study) manually segmented the same datasets used to train the classifier. The ‘Label Overlap Measures’ feature of the MorpholibJ plugin within ImageJ^24^ was used to compare the automatic and manual segmentations using the Sørensen–Dice coefficient, a commonly used metric,^25^, ^26^, ^27^, ^28^, ^29^, ^30^, ^31^ which ranges from 0 (no correlation) to 1 (identical images). This was found to be 0.963, indicating the automatic segmentation to be 96% similar to the manual segmentation (ground truth case). Due to the comparative sizes of the tubules and the microbranches, this validation was also performed on images containing only microbranches to prevent skewing due to higher accuracies on the larger tubule features. As the main tubules are much larger and occupy more of the classified volume, the automatic classifier is more likely to correctly identify these, giving a higher Sørensen–Dice score. It is important to ensure that the automatic classifier is also correctly identifying the smaller microbranches, and that these are not part of the 4% of dissimilar pixels. All objects on each slice, both tubules and microbranches were measured using the Analyze Particles function within ImageJ;^32^ there was an obvious change in object area between approximately 0.2–0.8 µm^2^, so this was used as the threshold: above this area were tubules, and below were microbranches. The tubules were removed from both the manually and automatically segmented images (using the Analyze Particles function, maintaining objects smaller than 0.5 µm^2^) and the same validation yielded a Sørensen–Dice coefficient of 0.754; a similarity of 75%. Despite being reduced, this value is still in‐line with acceptable values reported in literature, and so the use of the automatic classifier was accepted.^27^, ^29^, ^30^, ^31^

**FIGURE 2:**
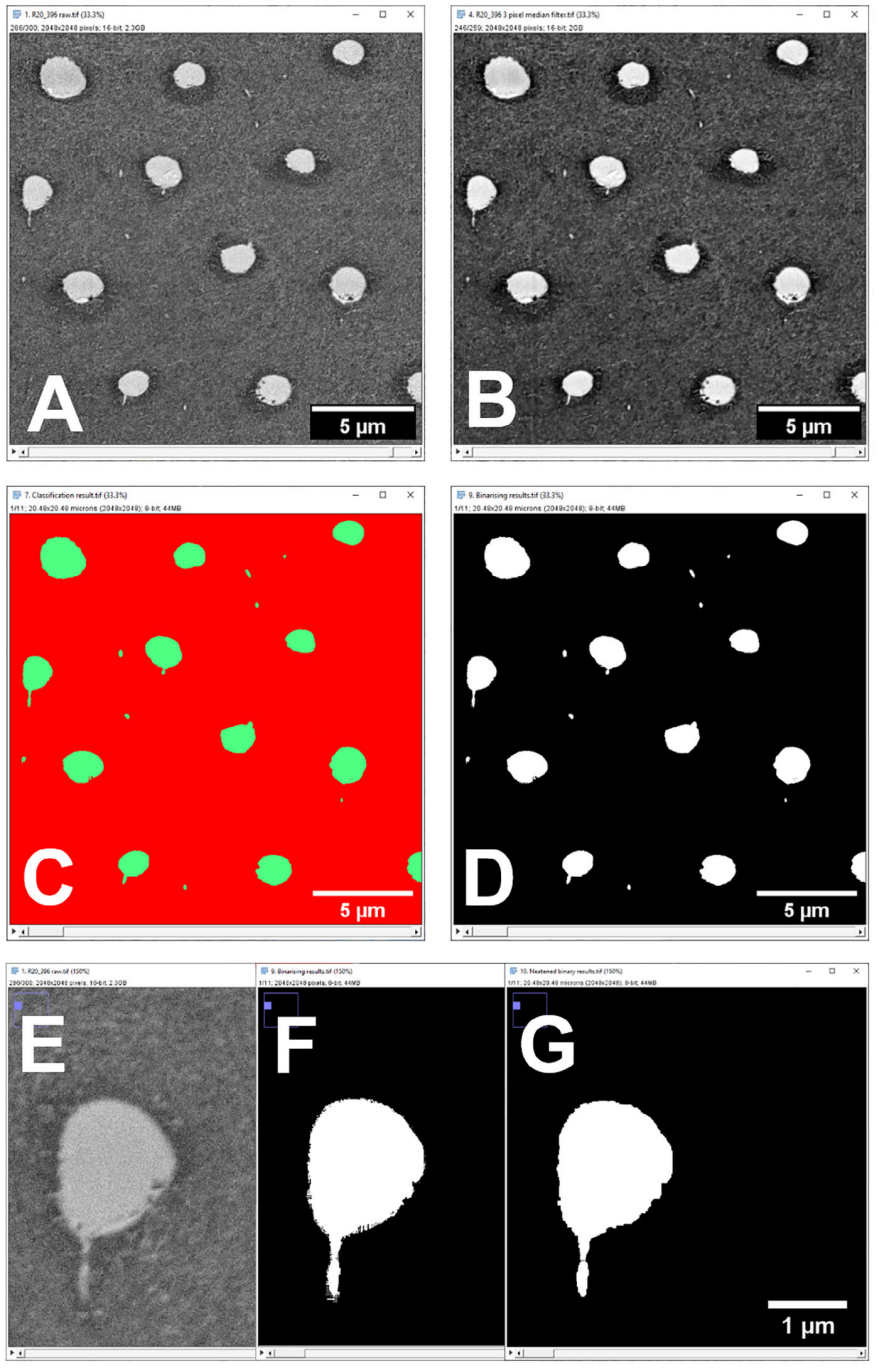
Workflow of image processing procedure: (A) Raw SEM image, (B) after adjusting contrast and applying 3‐pixel median filter, (C) after segmenting using classifier, (D) after binarising classifier results, (E) zoomed in raw image showing unclear tubule edge, (F) rough edges prior to neatening, (G) results after neatening with erosion and dilation.

The resulting binary images from the classifier had values of 0 (ITD) and 1 (tubules and microbranches). Some incorrectly classified stray particles were present, arising from the texture in the ITD (some noise remained after the application of the median filter); however these were only a small number of pixels. They were removed using equal number of erosion and dilation steps of the tubules and microbranches (up to a maximum of 3 for each operation, 6 in total), to ensure no overall change to feature morphology or size. This entire process, including the effects of the erosion and dilation can be seen in Figure 2.

### 2.4 Quantification of tubule and microbranch properties

In order to compare tooth locations listed in Table 1, five properties were identified and quantified in each area: tubule density, tubule diameter, microbranch density, microbranch diameter, and tissue porosity.

Tubule density was calculated by manually counting the average number of fully patent (open and unobstructed) tubules present on each slice, counted at 25‐slice intervals—a minimum of 12 slices was counted on the smallest stack, with an average of 20 slices counted overall. This was converted to a value per mm^2^ using the known field of view of the images (4.194 × 10^−4^ mm^2^). Tubule diameter was calculated as an ‘equivalent diameter’. This is a measure shown to be an accurate measurement method for irregularly circular shapes, similar to the tubule cross‐sections.^33^, ^34^, ^35^, ^36^, ^37^ Firstly, each dataset was rotated and resliced in ORS Dragonfly (version 2020.2),^38^ such that the tubules ran vertically through the stack. Then the area of each fully patent tubule was measured in ImageJ, at 100‐slice intervals. This area was then converted to an ‘equivalent diameter’ using the area of a circle equation.

Microbranch density was calculated by manually counting the number of microbranches present in each volume. The connectivity was also recorded by categorising microbranches into: (i) connected—both ends of the microbranch observed connecting to features, or (ii) unconnected—only one or neither end of the microbranch observed connecting to features. In the case where the microbranch was not segmented as a continuous feature (e.g. the segmentation was missing on some slices and therefore they do not appear visually continuous in the 3D space), the raw, unsegmented files were used to support accurate classification into (i) or (ii). This may have been a result of the binary operations (erosion and dilation) shown in Figure 2G removing very narrow sections of microbranch, incorrectly. However, the authors determined that the reduction in noise provided by these operations greatly outweighed this drawback, as the raw images could still be used on a manual case‐by‐case basis as a final confirmation. Due to the tortuosity and inconsistent directions of the microbranches, it was not possible to rotate and measure microbranch diameter in the same way as tubule diameter. Therefore, ellipses were fitted in ImageJ using the Fit Ellipse function,^32^ and the minor diameter of each ellipse recorded, to reduce the influence of any directionality of the microbranches, as they may not have been fully perpendicular to the imaging plane at the point of measuring.

In the binary processed images, the tubules and microbranches were white pixels, while the surrounding ITD was black. Tissue porosity was quantified by calculating the proportion of white pixels in each volume, both including and excluding the microbranches. Using the histograms of the binary images,^32^ the combined volume proportion of both tubules and microbranches (i.e., the number of white pixels) was recorded. Then, the Analyze Particles function^32^ was used to remove the smallest objects (the microbranches) and only leave the tubules remaining by filtering by object size and only keeping the largest objects. The total number of white pixels was then recorded again. Both values were presented as a percentage of the total image volume, with the difference accounting for the additional porosity by including the microbranches.

### 2.5 Normalisation

As well as comparing relative locations, the above quantities were also normalised by their distance from the pulp cavity. Previous literature in 2D has shown tubule geometry to change as a function of distance from the pulp cavity,^1^, ^5^, ^6^, ^12^ so it was hypothesised that microbranch geometry may also do so. The nearest distance from the pulp cavity to the centre of the sectioned sample was measured in ORS Dragonfly,^38^ and this was used to scale all figures from 0 (next to the pulp cavity) to 1 (at the dentinoenamel or dentinocementum junction). This removed any influence of varying tooth size, with different pulp cavity to DEJ distance. Results will be presented with respect to relative distance from the pulp cavity, using the scale defined above.

### 2.6 Statistical analysis

Statistical analysis was performed on all the results, except for microbranch diameter. For tubule density, tubule diameter, and microbranch density, a trend was determined by performing a simple linear regression to determine the coefficient of the regression line; a positive coefficient indicates an increasing trend, and vice versa. One‐way ANOVA tests were also used to determine significance between locations within the same tooth, and across all three teeth. For each region of interest, a pair of tissue porosity values was determined, one only including the tubules, and one including tubules and microbranches. These results failed a Shapiro–Wilk normality test (*p* < 0.050), and so a Wilcoxon Signed Rank Test was performed to compare these two values for each region of interest.

## 3 RESULTS

### 3.1 Tubule density

**FIGURE 3:**
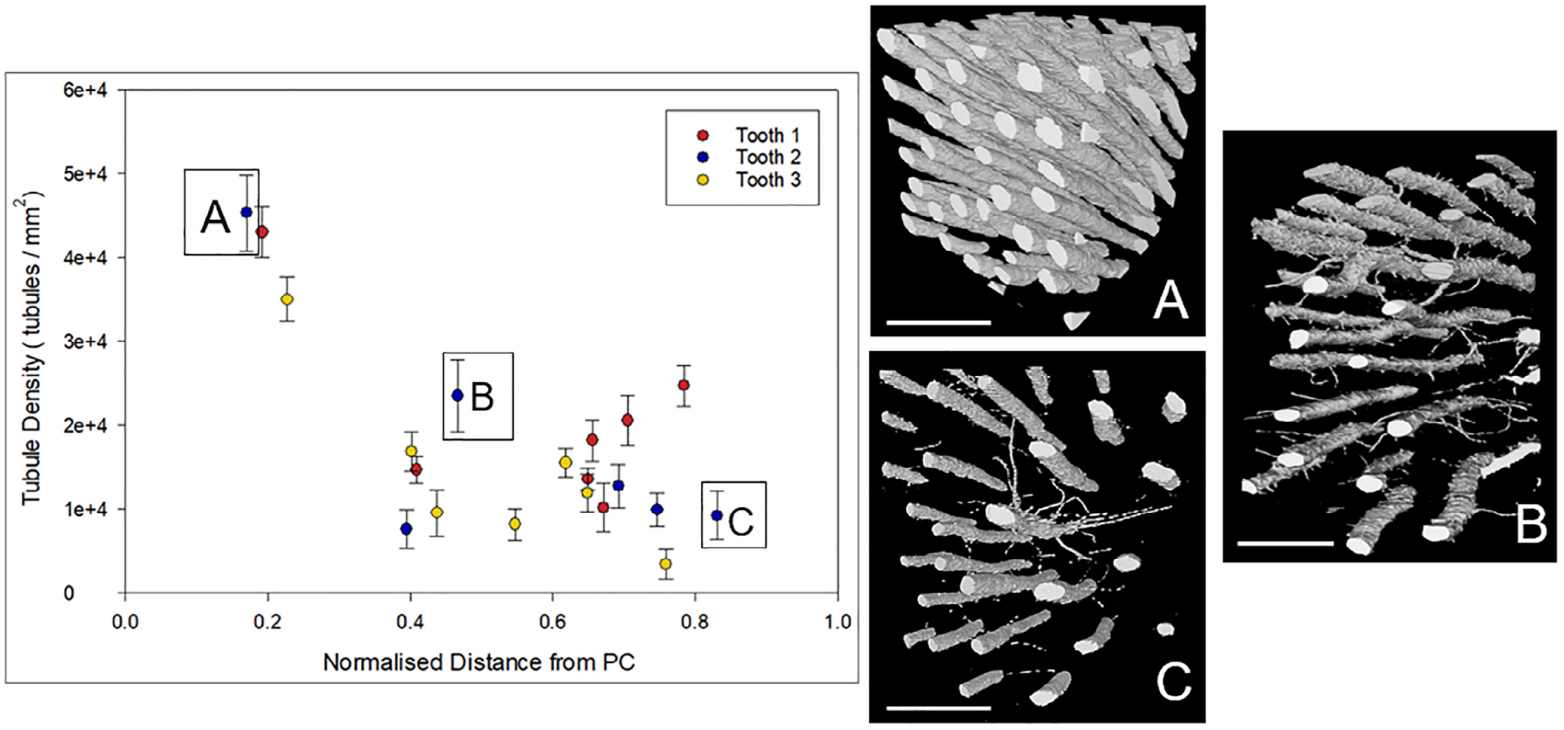
Average tubule density of each location (error bars = ± one standard deviation), scaled to tubules/mm^2^. *x*‐axis scaled such that 0.0 is the pulp cavity and 1.0 is the dentinoenamel or dentinocementum junction of each tooth. (A) Example of high tubule density found in the transitional pulp sample of Tooth #2. (B) Example of middle tubule density found in the upper root of Tooth #2. (C) Example of low tubule density found in under fissure of Tooth #2. White scale bar = 10 µm.

Tubule density for each region of interest is presented in Figure 3. There is a decreasing trend from a maximum density of 45 × 10^3^ tubules/mm^2^ at the pulp cavity, to 3.5 × 10^3^ tubules/mm^2^ at the DEJ, indicating that tubule density decreases further from the pulp cavity. A simple linear regression analysis found a coefficient of –38.9 × 10^3^, confirming a decreasing trend. It was observed that this trend lessens after a normalised distance of approximately 0.4; another regression analysis of only values after this point (i.e., closer to the DEJ) found a lower, albeit still negative coefficient of ‐6.7 × 10^3^. A one‐way ANOVA test reported statistical significance (*p* < 0.001) between each location in the same tooth. When each location was collated across all three teeth, *p* < 0.001 was also reported.

### 3.2 Tubule diameter

**FIGURE 4:**
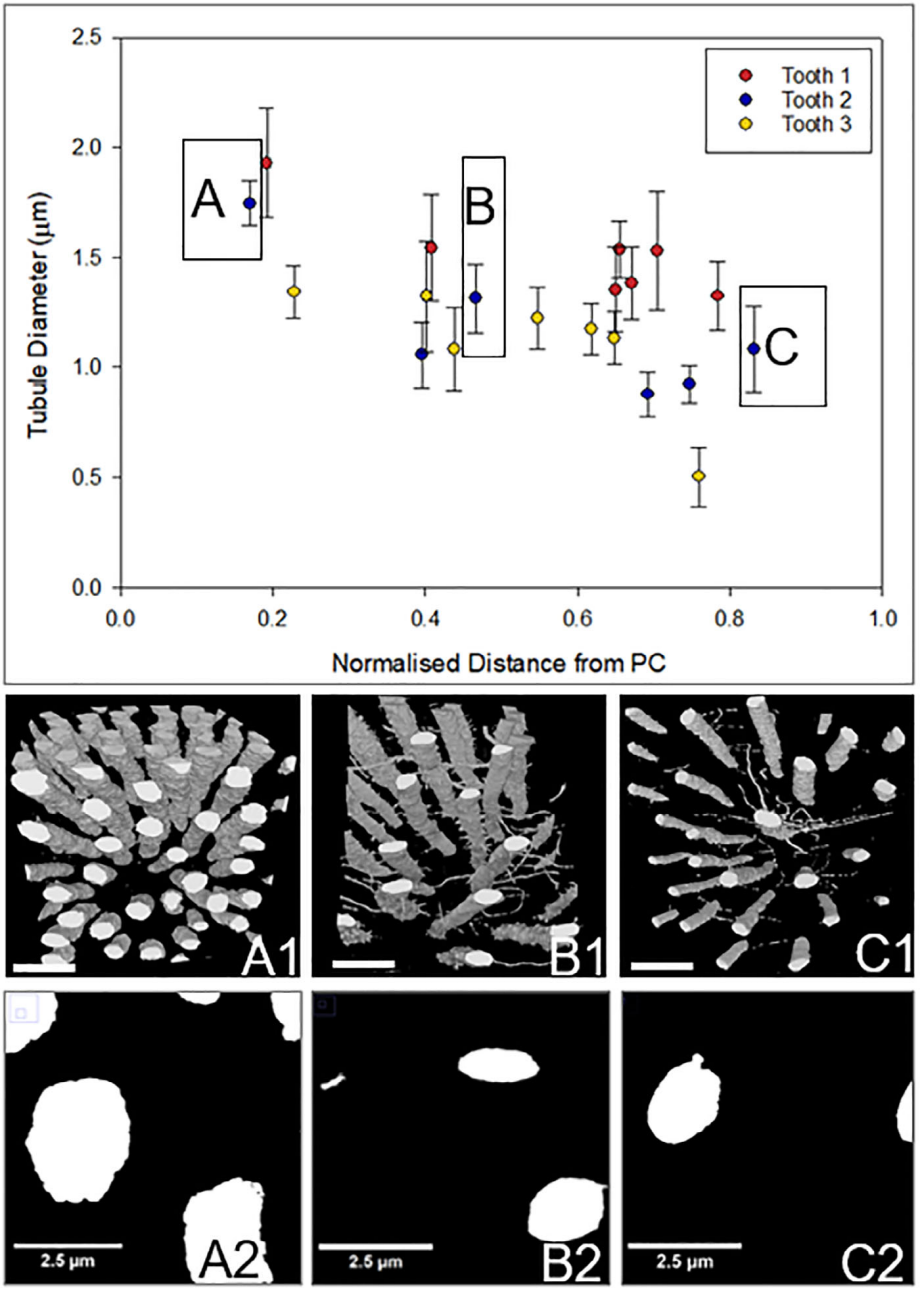
Average equivalent tubule diameter of each location (error bars = ± one standard deviation). *x*‐axis scaled such that 0.0 is the pulp cavity and 1.0 is the dentinoenamel or dentinocementum junction of each tooth. (A1, A2) Example of large tubule diameter found in the transitional pulp sample of Tooth #2, 3D representation and 2D cross‐section, respectively. (B1, B2) Example of middle tubule diameter found in the upper root of Tooth #2. (C1, C2) Example of small tubule diameter found in under fissure of Tooth #2, 3D representation and 2D cross‐section, respectively. White scale bar on A1, B1 and C1 = 5 µm. White scale bar on A2, B2 and C2 = 2.5 µm.

The average equivalent diameter for each region of interest is presented in Figure 4. Again, there is a decreasing trend from a maximum diameter of 1.93 µm at the pulp cavity down to 0.50 µm at the DEJ, indicating that the tubules narrow further away from the pulp cavity. A simple linear regression analysis found a coefficient of –0.89, confirming a decreasing trend. Similarly to tubule density, the coefficient decreases beyond a normalised distance of 0.4, down to –0.53. A one‐way ANOVA test reported statistical significance (*p* < 0.001) between each location in the same tooth. When each location was collated across all three teeth, *p* < 0.001 was also reported.

### 3.3 Microbranch density

**FIGURE 5:**
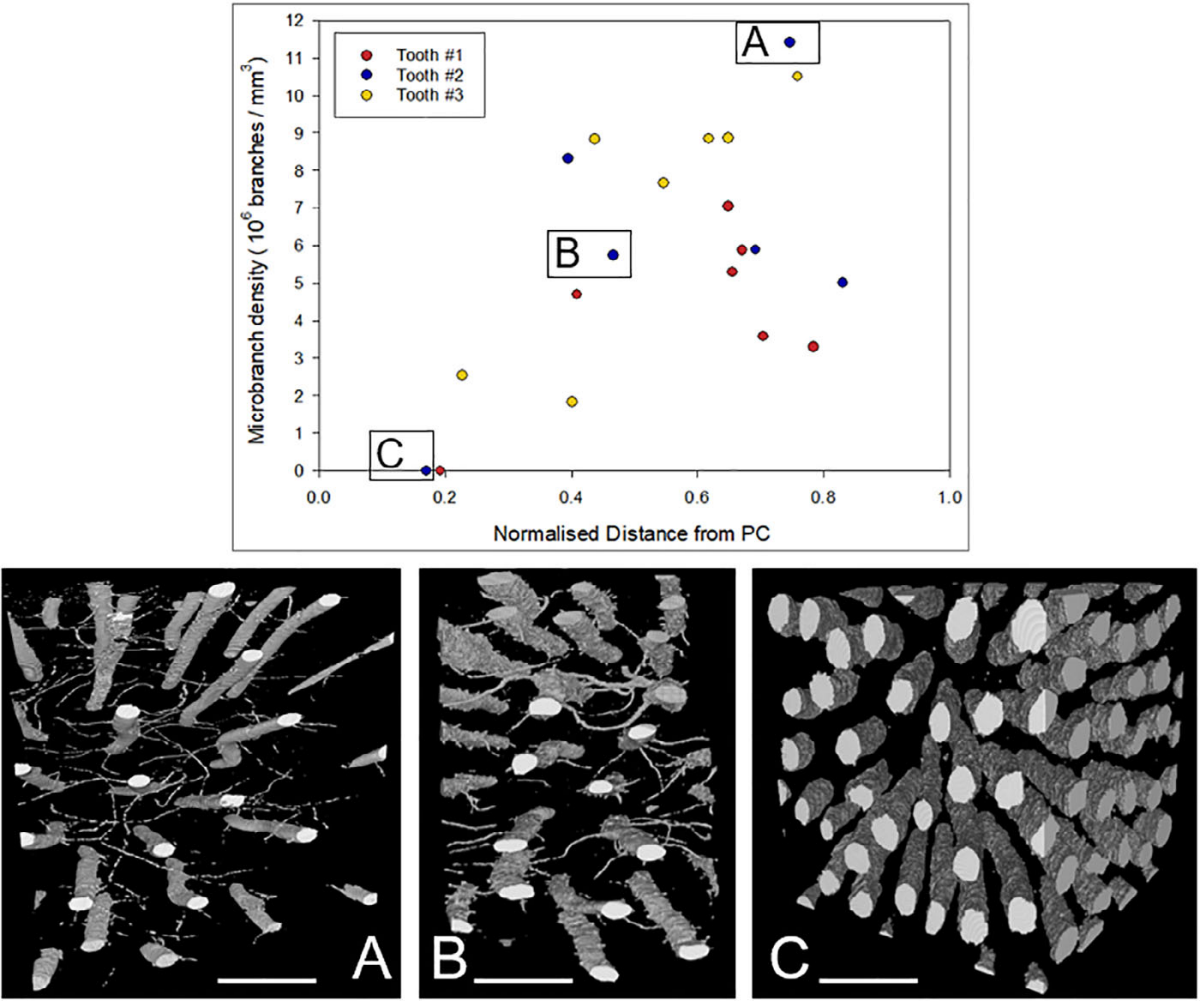
Microbranch density in each volume, scaled to 10^6^ microbranches/mm^3^. *x*‐axis scaled such that 0.0 is the pulp cavity and 1.0 is the dentinoenamel or dentinocementum junction of each tooth. (A) Example of high branching density found in under cusp of Tooth #2. (B) Example of middle branching density found in the upper root of Tooth #2. (C) Example of zero branching density, found in the transitional pulp sample of Tooth #2. White scale bar = 5 µm.

The measured microbranch density for each region of interest is presented in Figure 5. In contrast to the tubule diameter and density, this shows an increasing trend, indicating that there are more microbranches closer to the DEJ. A simple linear regression analysis confirmed this trend, with a coefficient of 8.9 × 10^6^. Two regions, both transitional pulp samples, showed no branching at all, while a maximum microbranch density of 1.14 × 10^7^ microbranches/mm^3^ was recorded under the biting cusp.

Only slight variations were observed in the percentage of microbranches fully connecting tubules (varying between 0 and 6%); however, this did not show any regional dependency. The majority of microbranches were seen to attach to a tubule, travel some distance into the ITD, but were unable to be traced further; this was either due to the microbranch appearing to terminate mid‐volume, or exit the imaged volume. A large portion of microbranches (between 23 and 45% of those classified as ‘unconnected’) could not be captured in their entirety as they were located to a certain extent outside of the imaged volume. While potentially resulting in overestimations of the amount of ‘unconnected’ microbranches, this has no effect on the measurement of the microbranch density or diameter.

### 3.4 Microbranch diameter

**FIGURE 6:**
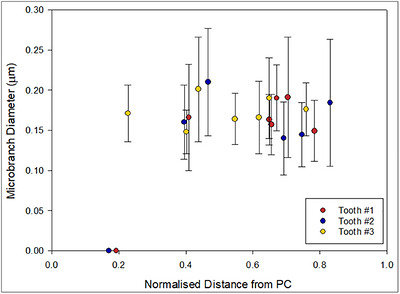
Average microbranch diameter of each measured location (error bars = ± one standard deviation). *x*‐axis scaled such that 0.0 is the pulp cavity and 1.0 is the dentinoenamel or dentinocementum junction of each tooth.

The average microbranch diameter for each region of interest is presented in Figure 6. Recorded diameter varied between an average of 140–210 nm, with an average standard deviation across all samples of 50 nm. No regional variation was observed between any sample locations.

### 3.5 Tissue porosity

On average, the volumes with only tubules had a porosity of 6.3%; however, this varied highly depending on location—one transitional periphery sample measured 0.8%, while another transitional pulp sample was 23.7%. When including the microbranches within these volumes, this average increased to 6.4% white pixels, an increase of 0.1% of the total volume, as shown in Table 2. While this change is small compared to the standard deviation for both of 5.7%, a Wilcoxon Signed Rank test comparing the porosity with and without microbranches for each region of interest showed statistically significant results (*p* < 0.001). A variation based on location was seen, with two transitional pulp samples showing no increase at all—as they had no branching—and others showing an up to 8% increase in the proportion of white pixels.

**TABLE 2:** Percentage of total volume pixels containing tubules only, and both tubules and microbranches, as well as the increase in volume porosity caused by inclusion of the microbranches, and the average porosity overall ± 1 standard deviation.

	Tissue porosity—tubules only (%)			Tissue porosity—tubules and microbranches (%)			
Location	Minimum	Maximum	Average	Minimum	Maximum	Average	Average increase (%)
Under cusp	2.73	4.29	3.31	2.84	4.38	3.42	0.10
Under Fissure	3.26	5.26	4.27	3.33	5.42	4.36	0.10
Bulk coronal	4.79	10.96	7.69	4.93	11.05	7.80	0.12
Transitional pulp	10.47	23.66	17.79	10.59	23.66	17.82	0.04
Transitional periphery	0.83	5.36	3.03	0.84	5.46	3.11	0.08
Upper root	2.09	6.22	3.61	2.26	6.37	3.76	0.16
Lower root	2.24	3.62	2.93	2.41	3.78	3.10	0.17
Average	Tubules only		6.25 ± 5.7	Tubules and microbranches		6.35 ± 5.7	

## 4 DISCUSSION

The use of SBF SEM in this study allowed for the generation of many high‐resolution, 3D images of both the tubules and microbranches within > 8000 µm^3^ volumes of dentine. While some small‐scale images of single microbranches, or similar (or larger) fields of view have been produced before, this is the first time that several regions of interest have been covered on a larger scale, with sufficient spatial resolution to quantify morphometric properties in 3D.^8^, ^11^, ^13^, ^39^, ^40^ FIB SEM is a comparable 3D imaging technique previously utilised for similar investigations, although previous studies have had larger slice thicknesses or been limited to smaller volumes and sample numbers.^13^, ^40^ One of the greatest benefits of both FIB‐ and SBF SEM is the superior resolution provided by the use of an electron microscope. Other non‐destructive 3D techniques, such as micro‐computed tomography, are able to scan larger volumes; however, they are limited by the achievable voxel size, which is often insufficient to resolve the smaller features such as microbranches.^8^, ^39^ A single sample with a larger field of view was imaged using CLSM by Vennat et al., albeit with lower spatial resolution (189 nm in‐plane, and 350 nm slice thickness)—this was sufficient to visualise and quantify the microbranching structure in the one location investigated.^11^

There were decreasing trends in both the density and the diameter of the main tubules on approach to the DEJ. This has been seen previously as a result of ‘tubule crowding’,^1^ as the tubules extend radially outwards from the pulp cavity. While the number of tubules between these two regions does not change, the equivalent surface area of the tissue does, forcing the tubules to appear further apart, giving the impression of a decreased density based on distance from the pulp cavity. The tubule density values found in this study are in line with those previously reported in literature, which range between 45 × 10^3^ and 76 × 10^3^ tubules/mm^2^ near the pulp cavity, dropping to between 19 × 10^3^ and 38 × 10^3^ tubules/mm^2^ at the DEJ.^1^, ^5^, ^8^ The large variations in these values within literature are likely a result of different sample preparation and imaging techniques, as well as inconsistent definitions of sample location (summarised simply as ‘inner’, ‘middle’ and ‘outer’ dentine), and natural variation between tooth geometries.

The imaging also demonstrated that the tubule diameter decreases with distance from the pulp cavity, in agreement with previous literature.^1^, ^6^, ^7^, ^8^ The values of tubule diameter (maximum diameter of 1.93 µm at the pulp down to 0.50 µm at the DEJ) are slightly smaller than those of the previous literature (1–4 µm);^6^, ^7^ however, the sample processing techniques in these previous studies are not clear. Other studies utilised decalcification prior to fixation and imaging, which may result in the loss of the peritubular dentine collar surrounding the tubule, resulting in an overestimated diameter value.^5^ It should be noted that no decalcification was done in this study, meaning the peritubular dentine remained intact, which would result in smaller measured tubule lumens. Other sources report lower values, varying linearly between 2.5 µm and 0.9 µm,^1^, ^5^ or approximately 1.75–2 µm diameters near to the DEJ.^9^, ^12^ These studies measured the diameter on 2D SEM images, which may explain the diametric increase if the tubule wasn't measured fully in cross‐section but as an ellipse, resulting in an overestimation of the diameter values. The linear trend in diameter is not as readily explained as the density variations—it is suggested that the peritubular dentine collar surrounding the tubule widens as dentine ages; therefore, there is more peritubular dentine present at the DEJ.^5^, ^9^, ^41^ During the process of dentinogenesis, the dentine begins mineralising at the dentinoenamel and dentinocementum junctions first, with the odontoblast processes withdrawing towards the pulp cavity.^1^, ^6^, ^11^, ^39^, ^42^ This would appear to support the trend that was found here, with additional peritubular dentine nearer to the DEJ causing the tubule diameter to narrow.

There appeared no trend or variation in the individual microbranch diameter observed as a function of location within the tooth (Figure 6). The majority of microbranches found in this study were below 0.2–0.3 µm in diameter, with some also recorded in the range of 0.3–0.7 µm, mostly under the occlusal fissure and in the root areas. These would be classified as ‘micro’ and ‘fine branches’ respectively by Mjör and Nordahl^10^ and are in line with the regional variations reported in previous literature. No microbranches larger than 0.7 µm were found, which is to be expected as these were observed in the peripheral 250 µm of dentine closest to the DEJ, which was not captured here.^10^ All measured microbranches exhibited a range of diameters spanning 50–470 nm, with an average diameter of 170 nm. The microbranch diameters per sample varied only by an average of 70 nm; this is considerably smaller than the variation observed in the tubule diameters (the smallest and largest recorded tubule diameters varied by 2.4 µm, with an average variation of 0.9 µm on individual samples). Some of this variation may also be an artefact of the measuring process. Unlike the highly oriented and directional main tubules, it was not possible to orient the image stack to be perpendicular to the microbranches due to their tortuosity. By instead recording the ‘minimum Feret diameter’, there is an assumption that there is no influence of microbranch drift or curvature causing an overestimation of size.^12^, ^43^

Conversely, the microbranch density showed the opposite trend to the tubule density and diameter, with an increasing density with distance from the pulp cavity, as shown in Figure 5. This agrees with previous findings.^10^, ^12^ The linear trend indicates an increase by a factor of four in the number of microbranches observed at the DEJ compared to at the pulp cavity. The increased microbranch density in regions of lower tubule density has the potential to influence the mechanics and fluid flow in these areas. Two of the three transitional pulp samples had zero branching, and consequently these regions showed both the highest tubule density and the largest tubule diameters. With the tubules occupying the majority of the volume porosity in all regions of interest, the influence of the microbranches on the fluid flow in areas of high tubule density and diameter would be reduced, which may be why the network is not as complex in these areas. Up to 6% of imaged microbranches were connected to other tubules, with a large portion of those not linking to the nearest neighbour but taking a tortuous path through the ITD and connecting to a tubule further away. There were also incidences of microbranches leaving and reconnecting back to the same tubule. From the current study it was not possible to identify any visual reason for the tortuous microbranch path through the ITD or any pattern to the microbranch connectivity.

An unpredicted limitation of this study was the chosen fields of view of the image stacks. Previous literature discussing any linkage or connectivity of microbranches refers to neighbouring or adjacent tubules;^9^, ^10^, ^12^, ^44^ however, the visualisations found in this study contradict this. Within the field of view captured, approximately one third of observed microbranches extended off the sides of the imaged volumes. They were consistently longer and more tortuous than expected. Some of these began in tubules near to the edge of the imaged volume already, while others travelled significant distances (> 5–10 µm) from nearer the centre of the image before reaching an edge, passing several tubules.

Previous literature has attempted to capture the contribution of the microbranches on the tissue porosity. Vennat et al. found that ‘fine branches’ accounted for more than 30% of all pore sizes within their measured volume—a non‐negligible contribution despite their relative smaller sizes compared to tubule diameters.^11^ This corresponded to an additional porosity in the tissue of approximately 0.4%.^12^ It should be noted that the chosen threshold used in this study on the CLSM images will have led to an overestimation of porosity by approximately 10%, as commented upon by the author.^11^ The average contribution of the microbranches to the tissue porosity was assessed here to be 0.1%; however, this varied highly by region—ranging from an additional 0.16% in the root, down to only 0.04% near the pulp cavity. While a similar order of magnitude to the values discussed by Hemmati,^12^ the results presented in this study appear lower and may be an effect of the imaging procedure used. As previously mentioned, the use of thresholding on the CLSM data will lead to an overestimation of tissue porosity, while others only assessed porosity as an area fraction on 2D SEM images.^9^, ^11^, ^12^ Despite these figures suggesting a low contribution, it should be noted that while the volume fraction of microbranches is low in comparison to the main tubules, their number and proliferation through the tissue is not, especially in areas of lower tubule density. The average microbranch diameter was significantly smaller (up to 10×) than the average tubule diameter, making their volume fraction appear lower. Their unexpected, highly tortuous nature made establishing a true value for connectivity difficult in the present work; however, it could be argued that this is a much more important metric for establishing their contribution to the tissue porosity. Having a small but highly connected network of microbranches connecting the main tubules may have large implications on the fluid flow within the tissue. Further discussions of the movement of the tubule fluid and the pulpo‐dentin complex are outside of the scope of this present study and have been discussed in great detail by others.^45^, ^46^ However, this work, and those performed by others discussed above have introduced an avenue for further investigation, and the inclusion of the non‐negligible contribution of the microbranches on the porosity of dentine tissue.

SBF SEM is not without imaging limitations; initial sample preparation relies on the penetration of a low‐viscosity resin, which is then polymerised. No vacuum was used for this process, only light centrifugation over several 6‐h periods. This may potentially have affected the resin penetration into the smallest ‘micro branches’, with diameters in the order of <200 nm, resulting in an underestimation of the microbranch density, the degree of connectivity, and the overall tissue porosity in this study. Other disadvantages of the technique include generating non‐isotropic voxels when imaging with a different pixel size and slice thicknesses, and the destructive nature of the technique due to the serial sectioning.

## 5 CONCLUSIONS

In conclusion, the initial goal of this study was to assess the morphological properties of the dentine microbranches in 3D, and assess regional variations of their size, structure and connectivity in the tooth. This was achieved through the use of SBF SEM to perform high‐resolution 3D imaging. The results found supported previous findings in literature, of a decreasing tubule diameter and tubule density moving from the pulp cavity to the DEJ, as well as showcased the microbranch network in higher resolution and on a larger scale than previously exhibited. Microbranch density was found to increase in areas of lower tubule density, while the diameter of these microbranches did not vary according to local anatomical location. These microbranches were found to be considerably more tortuous than previously discussed, connecting non‐neighbouring tubules, and travelling great distances through the imaged volume. Finally, quantification of the tissue porosity highlighted the contribution of this network, which also varied regionally around the tooth. By capturing the level of connectivity, microbranch density and the influence on porosity, this imaging can form the basis for investigations into the influence the microbranch network has on the tissue in terms of fluid flow and permeability.

## CONFLICT OF INTEREST STATEMENT

The authors declare no conflicts of interest.
